# From Regulated Cell Death to Adaptive Stress Strategies: Convergence and Divergence in Eukaryotic Cells

**DOI:** 10.1155/2018/2480462

**Published:** 2018-06-24

**Authors:** Sabrina Büttner, Paula Ludovico, Karin Thevissen

**Affiliations:** ^1^Department of Molecular Biosciences, The Wenner-Gren Institute, Stockholm University, 10691 Stockholm, Sweden; ^2^Institute of Molecular Biosciences, University of Graz, 8010 Graz, Austria; ^3^Life and Health Sciences Research Institute (ICVS), School of Medicine, University of Minho, 4710-057 Braga, Portugal; ^4^ICVS/3B's-PT Government Associate Laboratory, Braga/Guimarães, Portugal; ^5^Centre of Microbial and Plant Genetics (CMPG), Kasteelpark Arenberg 20, 3001 Heverlee, Belgium

Regulated cell death (RCD) encompasses different active forms of cell death ranging from apoptosis to necrosis or autophagy that are governed by distinct and highly sophisticated molecular pathways. An event intimately associated with RCD and RCD-associated pathologies, such as neurodegenerative or proliferative disorders, is redox homeostasis. A better understanding of how a cell responds to RCD induced by oxidative stress will undoubtedly uncover novel adaptive stress strategies, thereby providing more insights towards pro- or anti-RCD therapies.

This special issue unites 7 reviews and 5 original research articles. Various eukaryotic cell systems are discussed and include yeast, pathogenic fungi, zebrafish, and cancer cells. To uncover the complex network of the different and often interwoven RCD pathways and their association with oxidative stress, as well as for drug discovery purposes, lower eukaryotic cell model systems, including the budding yeast *Saccharomyces cerevisiae*, are used. In addition, due to the high conservation of RCD-related cellular processes, yeast can be used to study human pathologies caused by deviations in RCD. Moreover, the analysis of cell death routes solely present/induced in yeast and other fungi might point to novel therapeutic strategies to combat fungal infections.

To set the scene, K. F. Cooper describes the interplay between autophagy and apoptosis, thereby focusing in particular on the role of reactive oxygen species (ROS) in these processes. In addition, the molecular mechanisms underlying the induction and execution of RCD upon diverse types of stress and their association with oxidative stress have been addressed. Natural RCD-inducing scenarios such as ageing or disease have been considered as well as RCD induction upon challenge with external toxins. K. Mohammad et al. contributed with a relevant and timely review to the mechanisms of the liponecrotic mode of RCD induced by exogenous palmitoleic acid in the yeast *S. cerevisiae*. This review integrates liponecrotic RCD in different RCD scenarios, including age-related cell death modalities. In this respect, M. R. Yeaman et al. also provided a review on novel antifungal peptides that induce RCD in pathogenic fungi. Given the increasing impact of fungal infections on society due to the increasing emergence of resistance development and the relatively small armoury of antifungal compounds, antifungal agents that induce fungal cell death via a novel mode of action are of high relevance. Moreover, D. Wilkinson et al. contributed an original research article demonstrating that spatio-temporal metabolic differentiation within yeast colonies of different ages exists. They based their research on a comprehensive transcriptomic analysis and found that crucial metabolic reprogramming events arise during colony ageing, thereby supporting a role for mitochondria in this differentiation process. D. Wilkinson et al. also provided a review on the relevance of long noncoding RNAs (lncRNA) in differentiated subpopulations of yeast colonies and biofilms. Different cell types differ in their complements of lncRNA, which target diverse functional categories of genes in different cell subpopulations and specific colony types. These contributions highlight how *S. cerevisiae* colonies have become an excellent model for studying various aspects of microbial multicellularity, including cell differentiation and communication. In an original contribution, R. Zadrag-Tecza et al. used *S. cerevisiae* to study the impact of increased cell size on replicative ageing. An analysis of yeast cells harbouring differing genome copy numbers, ranging from haploid to tetraploid, provides evidence for a correlation between an increase in cell volume achieved via additional genome copies and the reproductive as well as the postreproductive lifespan.

Besides that, analysis of adaptive stress strategies or tolerance mechanisms elicited by different organisms under RCD stress or oxidative stress in general is of great interest. This knowledge can be exploited to develop effective combination therapy. In this respect, K. Vriens et al. contributed a study on the tolerance mechanisms that are induced by amphotericin B in yeast and in the human pathogen *Candida albicans* using a microfluidics platform. They show that the fungicidal action of amphotericin B can be increased by inhibiting the nitric oxide-dependent tolerance pathway via the NO synthase inhibitor L-NAME. This resulted in a fungicidal combination treatment based on AMB and L-NAME.

Redox homeostasis and cell death were also addressed in different diseases and models with therapeutic aims. M. Cobbaut and J. Van Lint discussed the role of protein kinase D (PKD) isoforms as regulators of the oxidative stress response. They highlight the differential activation of the specific PKD isoforms upon increased oxidative burden and the signaling pathways mediating their downstream effects as well as their isoform-specific characteristics. H. Gu et al. provided a review on the occurrence of radioresistance in gastric cancer, which is one of the primary causes responsible for therapeutic failure and recurrence of cancer and linked to ROS. In an original contribution, A. A. Kulkarni et al. used a transgenic nitroreductase-expressing zebrafish model treated with the prodrug metronidazole to demonstrate that ROS can be generated in a beta cell-specific manner. This hybrid chemical/genetic approach allows monitoring of ROS generation in beta cells in situ, and its manipulation, for instance by antioxidants, is shown to be protective for beta cells under conditions of elevated ROS production.

P. Davalli et al. contributed with a review on the DNA damage response (DDR) as a target for oxidative-based cancer therapy. DDR is deeply affected and sensitive to redox homeostasis since ROS regulate several enzymes of this repair pathway. The authors reviewed mechanisms contributing to ROS homeostasis, the ROS-sensitive proteins in DDR, and how DDR could be targeted in cancer therapy by using DDR inhibitors combined with ROS-inducing drugs. In an original article, S. V. Kostyuk et al. analysed the response of several human cell types to cell-free DNA differing in GC content and grade of oxidation. They provide insights into the different expression patterns of the transcription factors NF-*κ*B and NRF2 following treatment with distinct DNA species. While oxidized cell-free DNA elicited a very fast and intense inflammatory response, often leading to apoptosis, nonoxidized species rich in GC rather triggered a weaker response, promoting cell survival possibly via hormesis.

In conclusion, this special issue will provide more insights into RCD and into tolerance mechanisms elicited in cells experiencing RCD. Such information can prove very valuable in designing novel (combinatorial) pro- or anti-RCD therapies.

